# Prevalence of Noncarious Cervical Lesions and Gingival Recession in Subpopulations of Guinea-Bissau

**DOI:** 10.1155/ijod/8870847

**Published:** 2025-12-03

**Authors:** Paula Guerra da Rocha, Danna Mota Moreira, Antônio Sergio Teixeira de Menezes, Ana Karine Macedo Teixeira, João Victor de Paula Freitas, Jiovanne Rabelo Neri, Fabrício Bitu Sousa, Renata Mota Rodrigues Bitu Sousa, Smyrna Luiza Ximenes de Souza, Paulo Goberlânio de Barros Silva, Adriano de Aguiar Filgueira, Ramille Araújo Lima, Diana Araújo Cunha, Anya Pimentel Gomes Fernandes Vieira-Meyer

**Affiliations:** ^1^Department of Dentistry, University Center Christus, Fortaleza, Ceará, Brazil; ^2^Department of Clinical Dentistry, Faculty of Pharmacy Dentistry and Nursing, Federal University of Ceará, Fortaleza, Ceará, Brazil; ^3^Department of Dentistry, Unifacisa, Campina Grande, Paraíba, Brazil

**Keywords:** Africa, oral hygiene, tooth cervix, tooth wear, western

## Abstract

**Objectives:**

This study aimed to evaluate the prevalence of noncarious cervical lesions (NCCLs) and gingival recession (GR) in Guinea-Bissau and investigate their correlation with oral hygiene practices.

**Methods:**

Six calibrated examiners conducted intraoral examinations on individuals aged 12 years and above. A questionnaire gathered sociodemographic and oral hygiene information. NCCLs were identified through visual inspection of buccal and lingual/palatal tooth surfaces, while GR was assessed exclusively on buccal surfaces. The study adhered to World Health Organization (WHO) guidelines for oral health surveys.

**Results:**

A total of 233 participants took part, with 132 (56.9%) females and 100 (43.1%) males, of whom 165 (70.8%) lived in the capital and 68 (29.2%) on the Bijagós Islands. Age groups included 12–18 years (86; 36.9%), 19–34 years (67; 28.7%), and >34 years (80; 34.3%). GR was prevalent in 94.4% of the population, and NCCLs were present in 29.4%.

**Conclusions:**

There was no statistically significant difference in prevalence between the capital and the islands. The study did not find a significant association between NCCLs, GR, and oral hygiene practices. Nonetheless, both conditions showed correlations with age, gender, and treatment requirements, suggesting that individuals with GR are more predisposed to NCCLs.

## 1. Introduction

Guinea-Bissau is considered one of the 15 poorest nations in the world. With a life expectancy of 58 years, maternal mortality of 667/100,000 inhabitants and an extreme poverty rate of 40.7%, is classified by the United Nations Development Program as a nation with low human development index [1]. National reports show an average of 1.7 doctors for every 10,000 inhabitants, and still scarce the presence of specialized professionals, such as oral health professionals [1, 2]. Local sources indicate that there are only six dentists in the country.

The lack of data related to the oral health conditions of Guineans makes it impossible to understand their needs and organize adequate services for the promotion, prevention, and recovery of oral health in the country [3], limiting the development of appropriate policies for the population. Therefore, a survey of the oral characteristics of the population, encompassing the different age groups and regions of the country, is essential to ensure the development of processes and policies relevant to the population's needs. Considering the social, ethnic, and cultural diversities, it is important that easy-to-implement and valid methodologies are used in Guinea-Bissau, ensuring agility and comparability of results between different ages and regions, as well as with other areas of the world [3].

Among the conditions that need to be identified are noncarious cervical lesions (NCCLs) [4] and gingival recessions (GRs) [5, 6]. These two alterations are often linked to dentin exposure and hypersensitivity (HD) [6]. The mechanisms of action already identified as causing mineral tissue destruction in the cervical region in cases of NCCL refer to multiple contributing factors [7]. This multifactorial etiology is related to factors ranging from deleterious habits, parafunctions, the presence of temporomandibular dysfunction, and even the lifestyle of each patient [7, 8], with a growing prevalence in the young population [9].

Despite the heterogeneity of research data on the prevalence of NCCL around the world, to date there is no cataloged data on the prevalence of NCCL or GR in Guinea-Bissau. Therefore, the aim of this study was to investigate the prevalence of NCCL and GR in Guinea-Bissau, in relation to oral hygiene habits. The proposed study is relevant to public health, so that actions to prevent, mitigate, and recover from these alterations can be implemented, improving population's health and quality of life.

## 2. Methods

This cross-sectional observational research was submitted to the National Health Research Ethics Committee of Guinea-Bissau, regulated by the National Institute of Public Health, and approved under number 002/CNES/INASA/2023. In Brazil, it was approved by the Research Ethics Committee under number 6.166.163.

Data was collected in two administrative regions of Guinea-Bissau: Bissau and the Bijagós Islands. Data was collected in two villages located on Galinhas's Island and one village on Soga Island, and in three schools in the country's capital.

### 2.1. Sample and Sample Calculation

The sample consisted of 233 individuals, aged 12 or over. This study based its sample calculation on the research by Jordan et al. [10] carried out in Gambia, which has a population similar in number to that of Guinea-Bissau. Adopting a precision of 5% and a 95% confidence interval, a sample of 233 people was estimated.

The sample was collected by convenience, respecting the possibilities of insertion in communities with prior freedom of action for the researchers. However, they express part of the country's diversity by presenting data from the capital and noncapital, island versus mainland, variety of age groups, and diversity of ethnicities.

### 2.2. Characterization of Clinical Findings

The NCCL and GR data recording protocol is part of a broader survey, which included information on periodontal disease and caries. Data collection for the survey was based on the World Health Organization (WHO) oral health survey manual [11]. Data was collected by means of a questionnaire and a visual clinical examination [12] by six calibrated examiners. The patients were examined lying on long tables or benches, using clinical mirror and a WHO periodontal probe, under the illumination of a hand flashlight, directed by a trained assistant. All individuals aged 12 or over were included in the study. The exclusion criteria were edentulous patients. Visual inspection of the buccal palatal/lingual surfaces of all the teeth was carried out to detect the NCCL, while the GR was observed only on the buccal surfaces.

The clinical data collected was recorded on a clinical form adapted from the WHO [13]. GR was identified as present or absent, while NCCL was identified according to the severity index: “0” no sign of NCCL, “1” enamel lesion, “2” dentin lesion, and “3” pulp involvement. The prevalence of NCCL and GR was determined based on the absolute and relative frequencies of individuals with the alterations. The mean and standard deviation of the findings were determined for each individual and for each tooth. A specific questionnaire for the research, based on a WHO study [11], with sociodemographic data and oral hygiene data was applied by means of an oral interview, due to the high illiteracy rate in the populations studied.

NCCL was characterized by wear of the crown and/or root of the teeth in the cervical region, without the presence of caries [13]. Apical migration of at least 1 mm of the gingiva in relation to the cementoenamel junction characterized GR [14]. Both were determined by visual clinical inspection.

### 2.3. Calibration of Examiners

Data was collected by six previously calibrated professionals. The interexaminer calibration was carried out in nonface-to-face meetings, based on the methodology used by SBBrasil [15]. The interexaminer kappa value varied between 0.63 and 0.87. It is important to note that SBBrasil uses a kappa cut-off point of >0.61 to denote adequate training of its assessors [15].

### 2.4. Data Analysis

The data collected was analyzed using STATA 15.0 software and described by means and standard deviations (continuous variables) and frequency (categorical variables). To assess the relationship between variables, we used tests such as chi-square, *t*-student, one way ANOVA, and Pearson's correlation, depending on the type of variable (categorical or continuous) and the normality of continuous data (Kolmogorov–Sminorv test).

## 3. Results

The sample consisted of 233 individuals, 132 (56.9%) female and 100 (43.1%) males, of whom 165 (70.8%) were residents of the capital Bissau and 68 (29.2%) of the Bijagós Islands. One participant did not provide gender information in the survey. Data analysis was based on the categorical age groups of 12–18 years, 19–34 years, and over 34 years, and included 86 (36.9%), 67 (28.8%), and 80 (34.3%) people, respectively. The average age of the sample was 28.3 ± 14.9 years, and it was observed that the people assessed on the islands (33.6 years ± 16.4) were older than those in the capital (26.2 years ± 13.8) (*p*  < 0.05). The prevalence of GR found in this population was 94.4% (220 people) and that of NCCL was 29.4% (68 people) (Table 1). Some variables do not correspond to the total quantity due to the lack of information or even the participant's refusal to answer the survey for not wanting to inform or not having the information.

People from at least 13 different ethnic groups were assessed, the most frequent being Bijagó (*N* = 74; 33%), Balanta (*N* = 70; 31.2%), and Papel (*N* = 26; 11.6%). There was no difference in the prevalence of GR and NCCL between those living on the islands or in the capital (*p*  > 0.05).

With regards to the number of teeth in the mouth, 52.4% of the people examined had complete dentition, with 32 erupted teeth, 22.1% had 28 teeth, and 5.6% 24 teeth. The average number of teeth was 30.1, varying between 24 and 32 teeth, providing an assessment of more than 7000 teeth in this study. The descriptive results of the sociodemographic variables, oral hygiene practices, and dental needs are shown in Table 1, as well as the relationship of GR and NCCL with these variables.

Regarding the results for GR, a total of 233 people were assessed. GR was present in 220 individuals (94.4%) and 399 teeth. The most affected teeth were the upper molars (81; 35.06%) and the upper premolars (71; 30.74%), with a higher prevalence in the maxilla (210 teeth) than in the mandible (189 teeth). The average number of teeth with GR was 3.4 (SD ± 5.6), ranging from 0 to 30. There was a correlation between GR (*r* = 0.5128; *p*  < 0.001) and age, with a lower prevalence in the 12–18 age group. There was also a relationship between GR and the need for treatment (one face restoration) (*p*=0.005) (Table 1). There was a difference (*p*  < 0.05) in the mean numbers of teeth with GR between genders (women: 2.6 ± 4.3; men: 4.6 ± 6.7).

NCCL was assessed in 231 individuals and was present in 68 people (29.4%) of the sample in 222 teeth. The occurrence of NCCL increased with age, with a higher prevalence (43; 54.4%) in the >34 age group (Table 1). The most affected teeth were the upper and lower premolars (41.56%), with a higher prevalence in the maxilla (56.28%) than in the mandible (39.83%). Of the 68 individuals with NCCL who were examined, 44 (18.8%) had lesions with level 1 severity, 51 (21.9%) level 2, and 4 (1.7%) level 3. It is important to note that the same individual could have teeth with NCCL of different severities. There was a relationship between NCCL and gender (men had more NCCL than women). There was a trend in the relationship between NCCL and GR (chi-square; *p*  < 0.099), where individuals with GR tended to have NCCL.

Of the participants, 65.6% (153) were under 34 years old, and most (116; 66.7%) were studying. With regard to hygiene habits, 190 (81.5%) reported using a toothbrush, 162 (72.7%) brushed their teeth two or more times a day, and 205 (88.4%) used toothpaste. Other dental hygiene items were mentioned: 91 (39%) used charcoal, 24 (10.3.7%) fingers, and two (0.8%) twigs (Table 1). There was no relationship between dental hygiene practices and the existence of NCCL or GR. NCCL correlated (*p*  < 0.05) with age (higher in older people), gender (higher in males), schooling (higher in those who are not studying), need for dental treatment (higher in those who do not need treatment, and in those who need restoration of two or more faces or need extraction) and showed no relationship (*p*  > 0.05) with place of residence (capital or islands) or oral hygiene items (charcoal, twigs, toothbrush, and toothpaste) (Table 1). The frequency of NCCL presence by tooth group in the upper arch was incisors (20; 8.66%), canines (26; 11.26), premolars (48; 20.78%), and molars (36; 15.58%). The average for upper incisors was 0.2 (SD ± 0.7; range = 0–4); upper canines 0.16 (SD ± 0.5; range = 0–2); upper premolars 0.49 (SD ± 1.1; range = 0–4); and upper molars 0.27 (SD ± 0.7; range = 0–6). In the lower arch, the results were: incisors (14; 6.06%), canines (14; 6.06%), premolars (48; 20.78%), and molars (16; 6.93%). The average for lower incisors was 0.1 (SD ± 0.6; range = 0–4); lower canines 0.1 (SD ± 0.4; range = 0–3); lower premolars 0.4 (SD ± 1.0; range = 0–4); and lower molars 0.1 (SD ± 0.4; range = 0–4).

The need for dental treatment was identified in 192 (82.4%) individuals. In contrast, 41 (17.59%) individuals did not present the need for restorative, endodontic, and/or surgical treatment at the time of the clinical examination (Table 1). There was a significant increase (*p*  < 0.05) in the gradient of prevalence of need for treatment with increasing age for the variables need for two-faces or more restorative treatment and extraction. However, for the variables need for endodontics with restoration and one-face restorative treatment, the gradient increases from the 12–18 age group to the 19–34 age group but decreases from the 19–34 age group to the >34 age group (*p*  < 0.05), demonstrating that these needs increase up to a certain age and decrease after a certain period of life.

## 4. Discussion

This research is unprecedented in presenting oral health data in Guinea-Bissau, one of the poorest countries in Western Africa, with a huge shortage of human resources and minimal sanitary conditions. Epidemiological data makes it possible to draw up public policies for health promotion, prevention, and appropriate dental treatment [3]. The data presented in this population was not compatible with the global prevalence figures from systematic reviews, which indicate a 75%–85% prevalence of GR [16] and 46.7% of NCCL [17] in the world population. In the present study, the prevalence of GR was higher and that of NCCL was lower than the values presented worldwide. Hypotheses for this difference may be the age of those assessed (more than 65% are <34 years old), and the association of other sociocultural and dietary factors, which deserve further investigation. It is important to note the low number of tooth extractions and restorations, perhaps related to lack of access to oral treatment or difficulty in accessing sugar. However, this finding does not mean that preventive measures and/or oral hygiene instruction are not necessary. Studies have reported a relationship between NCCL and the elderly, urban dwellers, and those with higher incomes [12, 18]. In the country investigated, the similarity between the urban and rural populations may be due to the fact that, in Guinea-Bissau, in terms of people's general situation, regardless of their location, only a minority have access to better living conditions, food, and housing [1].

In the present study some variables do not correspond to the total quantity due to the lack of information or even the participant's refusal to answer the survey for not wanting to inform or not having the information. In some communities assessed, there were also difficulties in communicating or understanding the questions, leaving some variables unanswered.

Apart from cultural and communication challenges, comparing research is difficult. The diversity of terms and definitions, the lack of standardization and homogeneity of the data available in the literature, the variation in research methods and samples, as well as the different measurement indices are some of the factors that cause the results to fluctuate [17]. In Croatia, the prevalence of NCCL was 16.6% [19], while figures below 30% were found in seven other European countries [20]. However, in Bosnia and Herzegovina, the prevalence was 52%, with 16.3% in the 10–25 age group and 81.1% in people over 65, with no difference between genders [21]. The prevalence of NCCL in Guinea-Bissau was less than 30%. Even with lower values, NCCL should not be neglected, especially as it often starts at an early age [19].

Studies of NCCL in other populations around the world confirm our finding of a positive correlation between this lesion and age [20–23]. Savage et al. [20] found 60% of tooth wear lesions in Nigerian adults, with a positive relationship with brushing frequency. Authors suggest a correlation between NCCL and brushing items and techniques [22, 24].

In Guinea-Bissau, no correlation was found between NCCL and GR with oral hygiene items, nor was there any difference between residents in the capital or on the islands. Although items such as charcoal, fingers, and twigs are popularly used as means of oral hygiene, few interviewees admitted to using them. The majority of those interviewed said they used a toothbrush and toothpaste. Like Miller et al. [25], there was no association between brushing and the development of NCCL. Varenne et al. [26] assessed oral hygiene in Burkina Faso, covering both urban and rural areas, revealing that 58% of children and 35% of adults never brush their teeth and that a large part of the population uses chewing sticks for dental hygiene. As in Guinea-Bissau, Burkina Faso has a limited number of dental surgeons and poor access to health services [26].

In Guinea-Bissau, the occurrence of NCCL was positively related to age. The literature shows that NCCL are more prevalent in older patients [17, 21], and it should be considered that age shows the cumulative effect of various etiological factors of NCCL [12, 19].

Guinean men had a higher incidence of NCCL than women, similar to data from India, Poland, and China [18, 23, 27]. In contrast, research by Yoshizaki et al. [4] found a higher prevalence of NCCL in women (53%) than in men (47%) in Brazil. There are also studies that do not identify gender or age as factors related to the onset of NCCL [28]. However, the literature shows that gender and age, associated with other factors such as diet, place of residence, hygiene practices, and habits can influence the development of NCCL [27].

The results of this study showed a higher prevalence of NCCL in the maxilla than in the mandible, in agreement with studies carried out in Nigeria and Brazil [5, 26]. The most affected teeth were premolars, corroborating data from various countries [4, 12, 13, 18–21].

When NCCL involves the root and not just the crown of the tooth, GR is common [14]. Therefore, the correlation between NCCL and GR found in this study reiterates the findings of other authors [6, 29].

It is estimated that more than two-thirds of the world's population is affected by GR [16], which is more common in men, as confirmed in our study. Although cross-sectional studies do not allow assessment of the progression of GR, it is possible to state that not only GR, but also NCCL, tend to increase with age [6, 15, 18].

In Tanzania, the percentage of tooth loss in economically deprived individuals over 40 in rural areas was 93.2% [30], in contrast to the results found in Guinea-Bissau, where the majority of individuals have complete dentition and relatively low treatment needs/complexity. Although this result is intriguing given the lack of dental care and guidance and the precarious sanitary conditions and low income of most Guineans, it is important to consider that factors such as the age of the sample and uninvestigated dietary (e.g., low sugar intake) and cultural habits may interfere in this scenario and should be studied in the future.

The abrasiveness of the products used for dental hygiene poses a risk for the development of NCCL. Crushed charcoal, salt, and chewing sticks are abrasives widely used in Africa [31] but few Guineans reported using them in their dental hygiene, and there is no relationship between their use and the development of NCCL among them.

It's interesting to consider a few points that were observed empirically or ascertained informally by the researchers during the course of this research. The staple diet in the capital is rice with sauces made from “tcheben” (similar to palm oil) or “mancara” (peanuts) and, occasionally, fish. On the islands, food is basically rice and fish, but there are seasonal fruits such as mango, cashew and, occasionally, others such as banana, papaya, “mancubar,” “cabaceira,” “mampatas,” “farroba,” “veludo,” and “foles.” In general, the population of Guinea-Bissau eats only one meal a day, either in the morning or in the afternoon. The water used in the capital comes from wells, without any kind of treatment, while the drinking water on the islands comes from freshwater rivers or precarious wells.

Despite the relevance of the study, it was not without limitations. The convenience sample may lead to bias in the results. Regarding data reliability, it is important to mention that the questionnaire was answered by the subjects (self-report) and not by observation by the researchers. The complex logistics involved and the challenging conditions for carrying out each test and collecting the data, including the communication barrier (need for an interpreter, different dialects, and illiteracy), difficult access to the collection sites (especially the islands), are some of the many difficulties faced, not to mention the high cost of travel.

## 5. Conclusion

It was concluded that there is a high prevalence of GR (94.4%) and a relatively low percentage of NCCLs (29.4%) in the subpopulations of Guinea-Bissau. There was no difference between these conditions when comparing individuals living in the capital versus the islands, nor was there any relationship between oral hygiene habits and the development of NCCL and GR.

## Figures and Tables

**Table 1:** Descriptive data of sociodemographic variables, oral hygiene practices, dental needs of the Guineans investigated, and the relationship of the GR and NCCL with these variables.

		Patients with GR (*n* = 233)			Patients with NCCL (*n* = 231)		
	Total	No	Yes	*p*-Value	No	Yes	*p*-Value
Total	**233**	13 (5.56%)	220 (94.4%)	—	163 (70.6%)	68 (29.4%)	—
Location							
Capital	165 (70.8%)	8 (4.8%)	157 (95.2%)	0.449	114 (69.9%)	49 (30.1%)	0.747
Island	68 (29.2%)	5 (7.3%)	63 (92.7%)	—	49 (72.1%)	19 (27.9%)	—
Age							
12 to 18	86 (36.9%)	9 (10.5%)	77 (89.5%)	**0.045*⁣*^*∗*^**	83 (96.5%)	3 (3.5%)	**0.001*⁣*^*∗*^**
19 to 34	67 (28.8%)	3 (4.5%)	64 (95.5%)	—	44 (66.7%)	22 (33.3%)	—
>34	80 (34.3%)	5 (6.3%)	75 (93.7%)	—	36 (45.6%)	43 (54.4%)	—
Sex							
Female	132 (56.9%)	5 (3.8%)	127 (96.2%)	0.167	102 (77.9%)	29 (22.1%)	**0.005*⁣*^*∗*^**
Male	100 (43.1%)	8 (8%)	92 (92%)	—	60 (60.6%)	39 (39.4%)	—
Are you studying?							
No	58 (33.3%)	2 (3.5%)	56 (96.5%)	0.154	32 (55.2%)	26 (44.8%)	**0.001*⁣*^*∗*^**
Yes	116 (66.7%)	11 (9.5%)	105 (90.5%)	—	98 (85.2%)	17 (14.8%)	—
Frequency of cleaning teeth							
Never	1 (0.4%)	0 (0.0%)	1 (100%)	0.986	1 (50.0%)	1 (50%)	0.100
1x/month	2 (0.9%)	0 (0.0%)	2 (100%)	—	1 (50.0%)	1 (50%)	—
2–6x/week	2 (0.9%)	0 (0.0%)	2 (100%)	—	1 (50.0%)	1 (50%)	—
1x/day	56 (25.1%)	3 (5.4%)	53 (94.6%)	—	47 (85.5%)	8 (14.5%)	—
2x or more per day	162 (72.7%)	9 (5.6%)	153 (94.4%)	—	109 (67.7%)	52 (32.3%)	—
Hygiene items-toothbrush							
No	43 (18.45%)	2 (4.7%)	41 (95.3%)	0.769	29 (69.0%)	13 (31%)	0.812
Yes	190 (81.5%)	11 (5.8%)	179 (94.2%)	—	134 (70.9%)	55 (29.1%)	—
Hygiene items-finger							
No	209 (89.6%)	13 (6.2%)	196 (93.8%)	0.209	143 (69.1%)	64 (30.9%)	0.147
Yes	24 (10.3%)	0 (0.0%)	24 (100%)	—	20 (83.3%)	4 (16.7%)	—
Hygiene items-sanitizing branches							
No	231 (99.4%)	13 (5.6%)	218 (94.4%)	0.730	163 (70.9%)	67 (29.1%)	0.121
Yes	2 (0.8%)	0 (0.0%)	2 (100%)	—	0 (0.0%)	1 (100%)	—
Hygiene items-charcoal							
No	142 (60.94%)	8 (5.6%)	134 (94.4%)	0.964	103 (73.3%)	37 (26.4%)	0.213
Yes	91 (39.0%)	5 (5.5%)	86 (94.5%)	—	60 (65.9%)	31 (34.1%)	—
Use toothpaste							
No	27 (11.63%)	2 (7.4%)	25 (92.6%)	0.665	19 (73.1%)	7 (26.9%)	0.754
Yes	205 (88.4%)	11 (5.4%)	194 (94.6%)	—	143 (70.1%)	61 (29.9%)	—
Treatment required none							
No	192 (82.4%)	10 (5.2%)	182 (94.8%)	0.593	125 (65.8%)	65 (34.5%)	**0.001*⁣*^*∗*^**
Yes	41 (17.59%)	3 (7.3%)	38 (92.7%)	—	38 (92.7%)	3 (7.3%)	—
Treatment required one-face restoration							
No	110 (47.21%)	11 (10%)	99 (90%)	**0.005*⁣*^*∗*^**	76 (70.4%)	32 (29.6%)	0.952
Yes	123 (52.7%)	2 (1.6%)	121 (98.4%)	—	87 (70.7%)	36 (29.3%)	—
Treatment required two-face restoration or more							
No	141 (60.5%)	7 (5%)	134 (95%)	0.613	108 (77.1%)	32 (22.9%)	**0.006*⁣*^*∗*^**
Yes	92 (39.5%)	6 (6.5%)	86 (93.5%)	—	55 (60.4%)	36 (39.6%)	—
Necessary treatment endodontics restoration							
No	204 (87.5%)	12 (5.9%)	192 (94.1%)	0.593	144 (71.3%)	58 (28.7%)	0.524
Yes	29 (12.4%)	1 (3.5%)	28 (96.6%)	—	19 (65.5%)	10 (34.5%)	—
Treatment required extraction							
No	120 (51.5%)	9 (7.5%)	111 (89.5%)	0.188	91 (76.5%)	28 (23.5%)	**0.042*⁣*^*∗*^**
Yes	113 (48.4%)	4 (3.5%)	109 (96.5%)	—	72 (64.3%)	40 (34.7%)	—

*Note:* Some variables do not correspond to the total quantity due to the lack of information or even the participant's refusal to respond. The bold values indicate only the variables that are statistically significant, given the large number of values presented.

*⁣* ^*∗*^Statistically significant (*p* < 0.05).

## Data Availability

The data used in this study are available from the corresponding author upon reasonable request.
